# Resistance exercise exerts anti-hypertensive effects and downregulates NTPDase/CD39 and ecto-5′-nucleotidase/CD73 expression in patients with chronic kidney disease undergoing hemodialysis

**DOI:** 10.1007/s11302-025-10121-7

**Published:** 2026-01-21

**Authors:** Angela Makeli Kososki Dalagnol, Francini Franscescon, Matheus Chimelo Bianchini, Josiano Guilherme Puhle, Keroli Eloiza Tessaro da Silva, Helamã Moraes Santos, Sarah Franco Vieira de Oliveira Maciel, Débora Tavares de Resende e Silva

**Affiliations:** 1 Graduate Program in Biomedical Sciences, Federal University of Fronteira Sul (UFFS), SC 484 Highway, Southern Border, Chapecó, SC 89815-899 Brazil; 2 Graduate Program in Health Sciences, Universidade Comunitária da Região de Chapecó, Chapecó, SC Brazil

**Keywords:** ATP signaling, Platelets, Blood pressure, Purinergic system, Anti-hypertensive action

## Abstract

Chronic kidney disease (CKD) affects approximately 13.4% of the global population and significantly impacts patients’ quality of life. This study aimed to investigate the effects of resistance exercise on blood pressure and purinergic signaling in patients with CKD. Here, 28 patients with CKD performed a 12-week resistance exercise protocol during hemodialysis. Blood samples were collected before and after the intervention. Biochemical analyses, for example, NTPDases, ecto-5′-nucleotidase, and adenosine deaminase, were measured in platelets. Flow cytometry was performed to investigate CD39 and CD73 expression on lymphocytes. In addition, extracellular ATP and blood pressure were analyzed. Our findings revealed that patients with CKD present high systolic blood pressure (*p* = 0.0002) compared to control, and resistance exercise reduces blood pressure in these patients (*p* = 0.007). Regarding purinergic signaling, an increase in NTPDases, ecto-5′-nucleotidase, and adenosine deaminase was observed in patients with CKD (*p* = 0.0001; *p* = 0.0001; *p* = 0.0001; *p* = 0.0007, respectively). Surprisingly, after resistance exercise, NTPDase/ATP and ecto-5′-nucleotidase decreased (*p* = 0.0006; *p* = 0.02). CD39 and CD73 expression significantly increased on lymphocytes of CKD patients compared to control (*p* = 0.004; *p* = 0.0002, respectively). After resistance exercise, CD39 and CD73 expression was downregulated. Extracellular ATP levels were decreased in CKD (*p* = 0.0001), and resistance exercise restored these levels. In conclusion, CKD patients present high activity and expression of CD39 and CD73 enzymes, and resistance exercise mitigated purinergic exacerbation and presents anti-hypertensive effects in patients with CKD.

**Supplementary Information:**

The online version contains supplementary material available at 10.1007/s11302-025-10121-7.

## Introduction

Chronic kidney disease (CKD) is a global health problem characterized by the progressive and irreversible loss of normal kidney functionality [1]. It is known that the kidneys perform primary functions of filtering toxins, as well as metabolic waste products, and they are responsible for excretion and endocrine regulation. In cases of impaired performance of their functions, the condition of renal failure is established and can be classified as acute or chronic [2]. CKD is considered a complex condition involving non-modifiable risk factors (advanced age, genetic) and modifiable risk factors (diabetes, hypertension, dyslipidemia). These factors are responsible for the early onset of CKD as well as the progression of the disease [3]. While the course of kidney disease causes kidney failure or even death, people with CKD are more likely to die due to cardiovascular complications [4]. These complications are related to the modifiable risk factors mentioned above.

Purinergic signaling promotes a wide range of actions in the body, including cell migration and inflammatory responses. Purinergic receptors (P2Y_1_, P2Y_12_) and P2X (P2X_1_–P2X_7_), nucleotides (ATP, ADP, AMP), and enzymes (ectonucleotidases, adenosine deaminase) are widely distributed and found in leukocytes and platelets [5, 6]. NTPDase/CD39 is an enzyme expressed on the surface of the platelets and lymphocytes and hydrolyzes ATP to ADP and ADP to AMP, which is subsequently hydrolyzed into adenosine by ecto-5′-nucleotidase/CD73 [7, 8]. In platelets, the P2Y_1_ and P2Y_12_ receptors are specifically expressed, and ADP acts as an agonist promoting platelet aggregation [9]. The association of ectonucleotidases forms a cascade that acts to maintain some homeostatic process, e.g., preventing platelet aggregation because it regulates the extracellular nucleotide and nucleoside environment [10]. It is well known that platelet aggregation is induced by endothelial dysfunction [6]. Atherosclerosis is the leading cause of cardiovascular diseases, with both purinergic receptors and platelets playing key roles in this pathological process [11]. Therefore, inhibiting the overactivation of purinergic receptors in platelets may provide a protective effect on cardiovascular health. Some studies have been proposing that physical exercise plays a cardioprotective role [12–14]. Resistance exercise performed during 6 months has been shown to decrease thromboxane B2 (TXB2), ATP levels, AMP hydrolysis, ADP, and ATP hydrolysis in hypertensive women [12]. These findings suggest that resistance exercise can decrease platelet aggregation by modulating purinergic signaling in hypertensive women.

The benefits of physical exercise have been extensively demonstrated against a wide range of diseases and clinical conditions [12, 15–17]. On the other hand, a sedentary lifestyle may contribute to lower performance in physical activities, resulting in reduced muscle mass and strength [18]. Sedentary behavior is highly prevalent in CKD. It is well known that low levels of physical activity have a negative impact on quality of life and functional status and are strongly related to mortality and morbidity from the disease [19, 20]. Physical exercise can bring benefits by mitigating sarcopenia and improving cardiovascular health [21]. Resistance training, which involves few repetitions but is performed against moderate or high resistance, works large muscle groups and mainly impacts muscle mass and strength [21]. When performed for 12 weeks, resistance training was effective in recovering muscle strength and mass and decreased oxidative damage in patients with sarcopenia [22].

Previous studies indicate that resistance training can modulate metabolic parameters, e.g., increasing irisin levels [15], reducing blood pressure in hypertensive women [11]. To further explore the metabolic effects of physical activity during hemodialysis, we investigated its influence on metabolic markers, including myostatin and insulin-like growth factor (IGF), in CKD patients. Therefore, we decided to determine whether a resistance exercise protocol also exerts anti-hypertensive effects in CKD patients. Considering that purinergic receptors are present on platelets, and that platelet aggregation is present in stroke and acute myocardial infarction, assessing purinergic signaling on platelets is mechanistically justified. Therefore, this study aimed to measure the activity of purinergic enzymes, such as NTPDase (CD39) and ecto-5′-nucleotidase (CD73) as well as adenosine deaminase (ADA) in platelets of patients with CKD that performed a resistance physical protocol of 12 weeks undergoing hemodialysis. To further investigate the purinergic signaling in CKD as well as the effects of resistance exercise in these patients, we decided to measure extracellular ATP levels and assess the expression of CD39 and CD73 on lymphocytes using flow cytometry.

## Materials and methods

### Ethical statement

This is an interventional study with a quantitative approach of descriptive and comparative nature. It was reviewed and approved by the Institutional Ethics Committee of Federal University of Fronteira Sul (Chapecó, Santa Catarina, Brazil) under registration number (CAAE: 42603621.20000.5564). All procedures performed were in accordance with the relevant guidelines, as per the Declaration of Helsinki. Participants provided written informed consent prior to their inclusion in the study. Eligible patients were previously informed about the research and the use of biological material. The inclusion criteria were patients diagnosed with CKD on hemodialysis for at least 6 months, of both sexes, and over 18 years of age. Patients who adhered to less than 75% of the total sessions of the intervention protocol were excluded. At the start of the protocol, 36 patients were recruited. During the study, there were five dropouts, two deaths, and one transplant, resulting in 28 participants at the end of the experiment. In addition, 17 healthy controls were added in this study. Since this study does not constitute a clinical trial, clinical trial registration was not required (clinical trial number: not applicable).

### Intervention protocol

Resistance exercise protocol was performed and supervised by an exercise physiologist and a physiotherapist. Patients remain sitting or lying in a standard hemodialysis chair as previously described [23]. Each session lasted 45–50 min, consisting of 10–15 repetitions performed in 3 sets of each of the following exercises: (1) Extension of the knee and subsequent return (flexion) to the initial position; (2) hip flexion and subsequent return (extension) to the initial position; (3) hip abduction and subsequent return (adduction) to the initial position; (4) hip medial rotation and subsequent return (medial rotation) to the initial position; (5) flexion of the elbow and later return (extension) to the initial position; (6) elbow extension and subsequent return (flexion) to the initial position; (7) elevation of the scapula and subsequent return (depression) to the initial position; (8) shoulder abduction and subsequent return (adduction) to the starting position. Resistance exercise protocol was performed 3 times a week for 12 weeks, totaling 36 sessions. Depending on the location of the arteriovenous fistula located in the cubital fossa or the central venous catheter located in the jugular, subclavian, and femoral veins, exercises close to or involving that anatomical region were dispensed.

### Biological material collection

Blood samples were collected using tubes of 10 mL with ethylenediaminetetraacetic acid (EDTA) or separator gel and clot activator. Blood was collected before starting and after the end of the resistance exercise protocol. The samples were processed and fractionated into components (plasma, platelets, and leukocytes). The samples were processed and separated by centrifugation (5000 rpm, for 15 min, 20 °C). Biologic samples were stored in 1.5-mL microtubes at −80 °C for further analysis.

### Isolation of mononuclear cells and platelets from human blood

The buffy coat containing the white blood cells, located between the layer of plasma and erythrocytes, was carefully removed. The material was placed in a conical tube and diluted in an equal volume of saline solution. Subsequently, the diluted sample was transferred to a conical tube containing Lymphoprep (Ficoll-Histopaque), followed by centrifugation at 1800 revolutions per minute (rpm), at room temperature, for 30 min. After centrifugation, a density gradient was formed. In this way, there was the formation of an intermediate layer composed of mononuclear cells (lymphocytes and monocytes) between the layers of plasma and Ficoll. This cloud of cells was carefully removed with a Pasteur pipette over the upper layer (plasma) and transferred to a clean conical tube. Ten milliliters of saline solution were added to the cells, followed by centrifugation for 10 min at 1500 rpm. Thus, a lymphocyte concentrate was obtained. Mononuclear cells were used in further analyses.

To isolate platelets, the sample was first centrifuged at 1200 rpm for 10 min to separate erythrocytes and leukocytes; the supernatant (containing the platelets) was then carefully transferred to a new tube and centrifuged at 5000 rpm for 30 min, forming a platelet pellet at the bottom. After discarding the supernatant, the pellet was resuspended in 2 ml of HEPES buffer and subjected to a second centrifugation at 5000 rpm for 10 min to remove residual plasma proteins. If erythrocyte contamination persisted, a prior treatment with EDTA–ammonium chloride hemolytic buffer was performed before the final wash. Finally, the washed platelets were resuspended in a buffer. Platelets were used in further analysis.

### Analysis of the purinergic signaling

The NTPDase activity was measured in platelets. A colorimetric assay was performed to measure the amount of inorganic phosphate (Pi) released. A volume of 20 µL of platelet sample was mixed alone with 160 µL of the NTPDase incubation system and pre-incubated for 10 min at 37 °C. The incubation system for NTPDase contained 1200 mM of NaCl, 600 mM of glucose, 50 mM of KCl, 500 mM of Tris-HCl pH 7.4, 50 mM of CaCl_2_, and ultrapure water. The reaction was initiated when 20 µL of substrate (ATP or ADP) was added. NTPDase activity was stopped by adding 150 µL of 15% trichloroacetic acid (TCA). Readings were taken on a spectrophotometer at 630 nm. All samples were analyzed in triplicate. The control of non-enzymatic hydrolysis was carried out using an identical procedure to that of the samples, but without the addition of enzymes. A standard curve was prepared with 1 mM of KH_2_PO_4_. The results of enzymatic activity were expressed in nmol of Pi released per minute per milligram of protein (nmol Pi/min/mg protein). The ecto-5′-nucleotidase activity was quantified in platelets as previously described [12, 24, 25]. Adenosine deaminase (ADA) activity was performed based on the measurement of ammonia produced following a previous produced method [25, 26]. ADA activity was determined using 30 µL of platelets and 90 µL of adenosine substrate (21 mM–pH 6.5). The reaction was incubated at 37 °C for 60 min. Subsequently, 80 µL of phenol/sodium nitroprusside and 80 µL of alkaline hypochlorite (12.5 mL of 1 M NaOH in 1.6 mL of 5% alkaline hypochlorite in 100 mL of H₂O) were added to the solution. The mixture was incubated at 37 °C for 30 min. Absorbance was measured at 620 nm. The results were expressed in ADA units per liter (U/L). ATP extracellular levels were measured using a commercial kit for ATP *Determination* (Invitrogen®), following the manufacturer’s instructions.

### NTPDase/CD39 and ecto-5′-nucleotidase/CD73 expression

Flow cytometry analysis was performed to investigate NTPDase/CD39 and ecto-5′-nucleotidase/CD73 expression in lymphocytes of patients with CKD undergoing hemodialysis as previously described [27]. Monoclonal antibodies (anti-CD39) APC (FITC) and (anti-CD73) NTPDase (PE) were used in this study. Briefly, lymphocytes were resuspended in saline solution. Cells were counted in a Neubauer chamber, and a total of 10^6^ cells were considered suitable for analysis. A total of 100 µl of lymphocyte suspension was incubated with 20 µl of anti-CD39 and 5 µl of anti-CD73 at 4 °C for 30 min. The mix was centrifuged at 200 g for 3 min (2–8 °C), and the supernatant was discarded. The pellet was washed with 200 µl of buffer/saline solution. The final mix (200 µl) was added to a U-button 96-well plate. After staining, the cells were acquired (10,000 events) using a BD Accuri™ C6 Plus flow cytometer (San Diego, CA, USA). Data were obtained by analyzing the events based on cell size parameters (FSC), granularity (SSC, Side Scatter), and fluorescence intensity in the respective detection channels. These were analyzed and subsequently compared with an unstained control. Finally, the results were expressed as the percentage of stained cells relative to the unstained control and statistically analyzed to assess ectonucleotidase expression.

### Protein quantification

Protein levels in plasma and platelets were determined by the Coomassie blue method using bovine serum albumin as the standard [28]. Absorbance of samples was measured at 595 nm.

### Statistical analysis

The normality and homoscedasticity of data were analyzed by the Shapiro-Wilk test. To analyze blood pressure, heart rate, and ectonucleotidase activity, one-way ANOVA was applied, followed by Tukey post-hoc multiple comparisons test whenever necessary. The Kruskal-Wallis test followed by Dunn’s post-hoc test was used for non-parametric data. IGF-1 and myostatin levels in CKD were analyzed by Mann-Whitney test. Results were expressed as median with interquartile range or mean and SD. Differences were considered statistically significant when *p* ≤ 0.05.

## Results

### Sample characterization

Patients on a regular hemodialysis program (28 individuals, 14 men/14 women) and 16 healthy controls participated in this study. Participants had a mean age of 49.70 ± 16.77 years. The healthy control group in the present study consisted of 10 women and 7 men, aged between 31 and 64 years. Both demographic data of our healthy control and CKD patients are shown in (Table 1). In addition to CKD, many patients presented with comorbidities which are highlighted in Table 1. These comorbidities included hypertension (92.85%), DM type II (25%), hypothyroidism (3.57%), and hyperthyroidism (3.57%). All patients with type II DM had hypertension. Table 1 also lists the medications used by CKD patients, including antihypertensives, insulin, levothyroxine, analgesics, gastrointestinal medications, central nervous system (CNS) medications, iron hydroxide, and erythropoietin. Only a small proportion of patients (7.14%) were using statins, specifically simvastatin. Additionally, many patients were taking calcium and vitamin supplements (data not shown). Regarding sample characterization, around 60% of patients with CKD were non-smokers and non-alcohol users. However, for 40% of the patients, information on smoking and alcohol consumption was not provided. Importantly, systolic blood pressure and creatinine levels were decreased following resistance exercise (*p* = 0.008; *p* = 0.0247, respectively). Blood parameters including platelet count, leukocyte count, hematocrit, and hemoglobin levels were assessed and showed no changes after resistance exercise.

**Table 1:** Sample characterization of healthy control and patients with CKD which performed a resistance exercise protocol. Patients’ medical treatment (e.g., insulin dependents, hypertensive, and hypothyroidism are highlighted)

Sample characterization							
**Control patients**							
Total of sample	Group = 17						
***Sex***	***Nº***	***%***	***BMI***	** < *18.4***	***18.5–24.9***	***25–29.9***	** > *30***
*Male*	7	41%	30.68	0	3	2	2
*Female*	10	59%	27.10	0	3	5	2
			***Age***	***Smokers***	***Alcoholics***		
				*No*	*Not informed*		
*% of male*			47.67 ± 7.28	7 (100%)	-		
*% of female*					-		
			37.29 ± 10.37	10 (100%)			
			***% of total***	**17 (100%)**	**-**		
***Comorbidities***			***Hypertension***	***DM II***	***Hypothyroidism***	***Hyperthyroidism***	
			3 (17.64%)	1 (5.88%)	-	-	
***Medicine***			***Anti-hypertensive***	***CNS medications***	***Hypoglycemic***		
*% of male*			1 (5.88%)	-	1 (5.88%)		
*% of female*			2 (11.76%)	3 (17.64%)	-		
***% of total***			**3 (17.64%)**	**3 (17.64%)**	**1 (5.88%)**		
**CKD Patients**							
Total of sample	Group = 28						
***Sex***	***Nº***	***%***	***BMI***	** < *18.4***	***18.5–24.9***	***25–29.9***	** > *30***
*Male*	14	50%	27.5	0	6	2	6
*Female*	14	50%	23.9	1	5	8	0
			***Age***	***Smokers***	***Alcoholics***		
				*No*	*Not informed*		
*% of Male*			49.15 ± 15.42	8 (54.14%)	6 (42.85%)		
*% of Female*			50.21 ± 18.50	9 (64.28%)	5 (35.71%)		
***% of total***				**17 (60.71%)**	**11 (39.28%)**		
***Comorbidities***			***Hypertension***	***DM II***	***Hypothyroidism***	***Hyperthyroidism***	
			26 (92.85%)	7 (25%)	1 (3.57%)	1 (3.57%)	
***Medicine***			***Anti-hypertensive***	***Insulin***	***CNS medications***	***Analgesic***	
*% of Male*			14 (100%)	4 (28.57%)	10 (71.42%)	7 (50.00%)	
*% of Female*			12 (85.71%)	3 (21.42%)	10 (71.42%)	7 (50.00%)	
***% of total***			**26 (92.85%)**	**7 (25%)**	**20 (71.42%)**	**14 (50.00%)**	
			***Levothyroxine***	***Iron-hydroxide***	***Erythropoietin***	***Gastrointestinal medications***	
*% of male*			0 (0%)	14 (100%)	14 (100%)	13 (92.86%)	
*% of female*			4 (28.57%)	14 (100%)	14 (100%)	13 (92.86%)	
***% of total***			**4 (14.3%)**	**28 (100%)**	**28 (100%)**	**26 (92.86%)**	
			***Statins***				
*% of male*			1 (7.14%)				
*% of female*			1 (7.14%)				
***% of total***			**2 (7.14%)**				
***Creatinine mg/dL***							
			**Before**	**After**			
*Male*			11.23 ± 2.67	8.99 ± 2.61			
*Female*			8.881 ± 2.742	8.053 ± 3.071			
*Total*			10.19 ± 2.85	8.411 ± 2.72			

Data are expressed as mean ± SD or by percentage (%). Values to creatinine were considered before and after resistance exercise protocol

*BMI* body mass index

^*^Significance after resistance exercise: *p < 0.05 and **p < 0.01

#### Physical exercise decreases blood pressure and modulates metabolic endpoints in patients with CKD

**Fig. 1:**
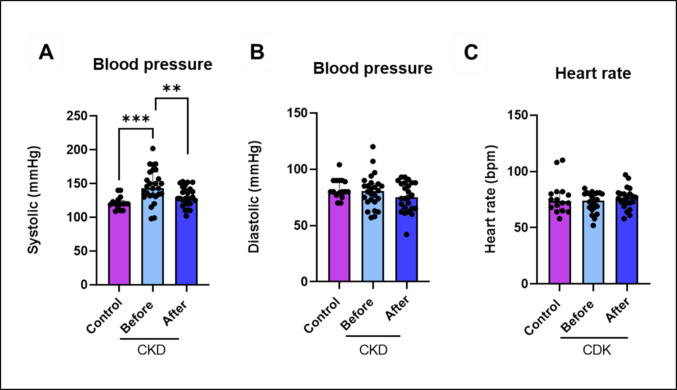
Data were analyzed by one-way ANOVA followed by Tukey’s multiple comparisons test. Results are expressed as mean and SD. Asterisk (*) indicates a significant difference: *p < 0.05 and **p < 0.001 (*n* = 16–28)

Effects of physical exercise on blood pressure and heart rate of CKD patients and healthy controls (Fig. 1). One-way ANOVA yielded that CKD patients present significative high blood pressure compared with control (*F*_(2,67)_ = 10.07; *p* = 0.0002). Interestingly, resistance exercise decreases systolic blood pressure in CKD (*p* = 0.007), while diastolic blood pressure remains unchanged. Heart rate did not change when compared CKD and healthy controls. Furthermore, when compared with healthy controls in the literature, IGF-1 and myostatin levels of CKD patients were lower (Supplementary material Table 1), and resistance exercise increases IGF-1 (*p* = 0.007) and reduces myostatin (*p* = 0.04) in CKD patients.

#### Ectonucleotidase activity and extracellular ATP levels are altered in patients with CKD, and these responses are modulated by resistance exercise

**Fig. 2:**
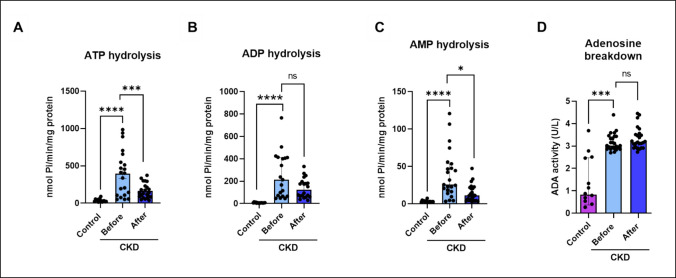
Ectonucleotidases activity analyzed in platelets of patients with CKD submitted to the physical exercise protocol. In platelets, NTPDase/CD39 activity in relation to ATP hydrolysis (**A**), NTPDase/CD39 activity in relation to ADP hydrolysis (**B**), ecto-5′-nucleotidase/CD73 activity in relation to AMP hydrolysis (**C**), ADA activity (**D**). Data were expressed as median and interquartile range or S.E.M. The results were analyzed by one-way ANOVA (ATP hydrolysis) or by the Kruskal-Wallis test. Asterisk (*) indicates statistically significant differences: *p ≤ 0.05, ***p ≤ 0.001, and ****p ≤ 0.0001 (*n* = 14–28)

**Fig. 3:**
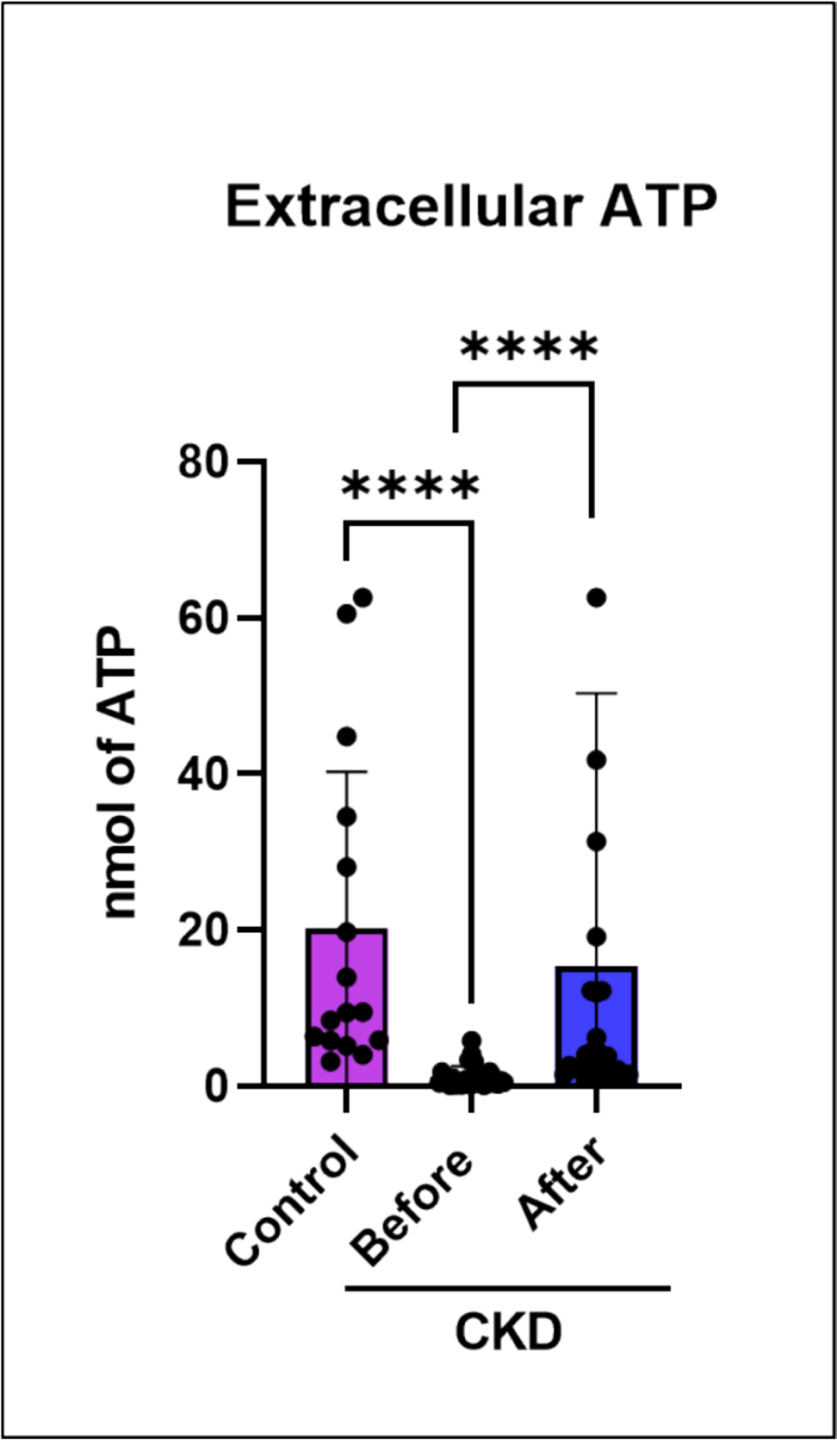
Extracellular ATP levels in patients with CKD. Patients with CKD exhibited reduced extracellular ATP levels compared to control. Interestingly, after resistance exercise extracellular ATP levels were similar to those observed in the control group. Data were analyzed by Kruskal-Wallis test and expressed as median and interquartile range. *Significance statistic was accepted when ****p ≤ 0.0001 (*n* = 16–28)

Ectonucleotidases activity on platelets of patients with CKD and in healthy controls (Fig. 2). One-way ANOVA yielded that NTPDase/ATP increased in CKD patients (*F*_(2, 56)_ = 16.43; *p* = 0.0001). Kruskal-Wallis test indicated that NTPDase/ADP, ecto-5′-nucleotidase and ADA also increased on platelets of patients with CKD when compared to control (*p* = 0.0001; *p* = 0.0001; *p* = 0.0007, respectively). On the other hand, patients with CKD that performed a resistance exercise decreased ATP hydrolysis (*p* = 0.0006). After physical exercise, AMP hydrolysis was decreased in CKD patients (*p* = 0.02). Adenosine breakdown did not change after physical exercise. Interestingly, extracellular ATP levels were significantly decreased compared to the control group (*p* = 0.0001) (Fig. 3), while ATP levels were restored after physical exercise (*p* = 0.0001) in CKD patients.

#### Physical exercise reduces CD39 and CD73 expression on lymphocytes of patients with CKD

**Fig. 4:**
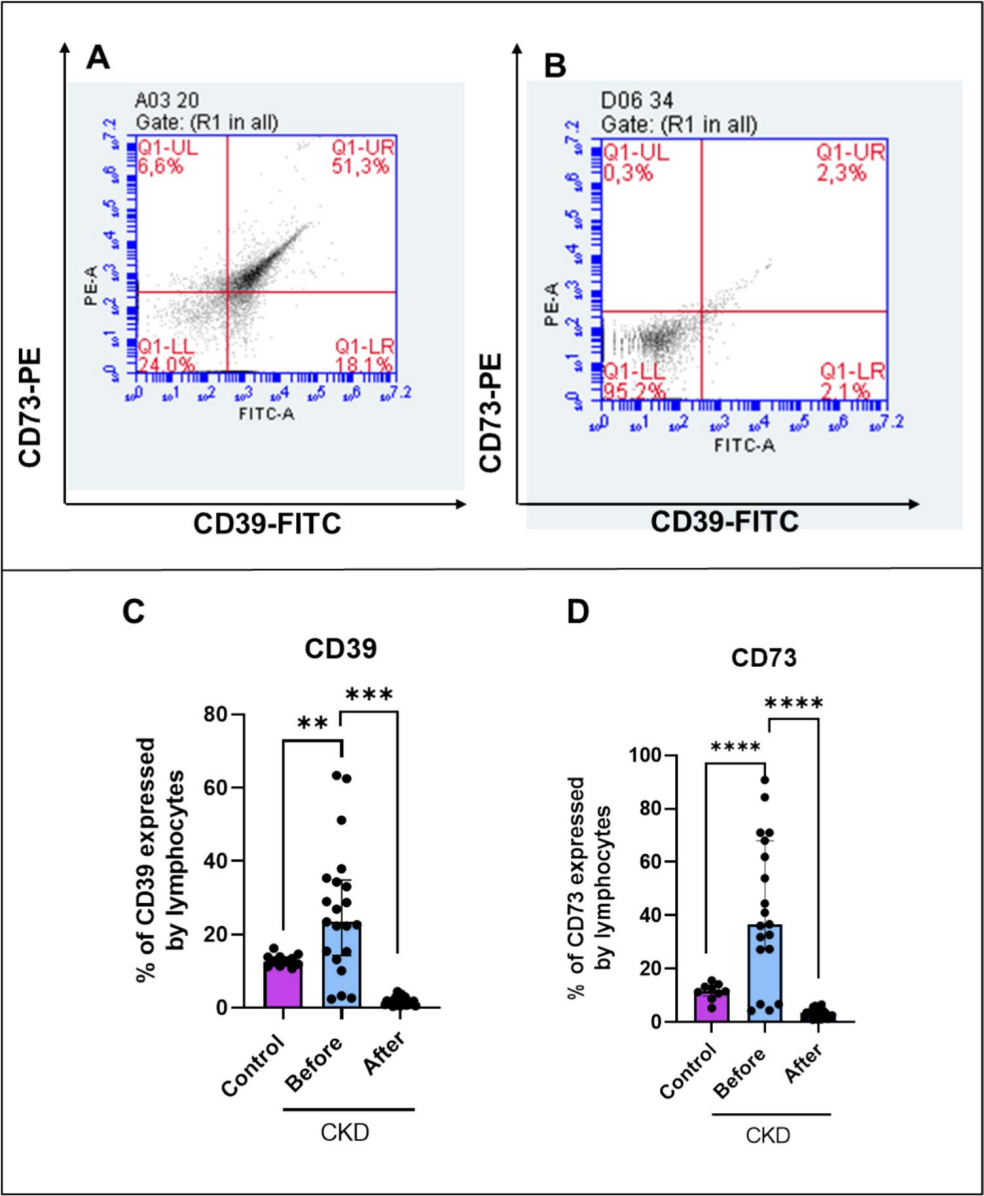
Representative flow cytometry dot-plots of patients with CKD submitted to resistance exercise. **A** Before and **B** after resistance exercise in lymphocytes of patients with CKD. **C** CD39 and **D** CD73 expression. Data were expressed as median and interquartile range. Asterisk (*) indicates a significant difference: *p ≤ 0.05, **p ≤ 0.01, ***p ≤ 0.001, and ****p < 0.0001 (*n* = 9–20)

In addition to assessing ectonucleotidase activity, we analyzed the expression of CD39 and CD73 in lymphocytes of patients with CKD undergoing hemodialysis and healthy controls. One-way ANOVA yielded that CKD patients present significative high levels of CD39 (*F*_(2, 52)_ = 28.64; *p* = 0.004) and CD73 (*F*_(2, 45)_ = 26.60; *p* = 0.0002) expression on lymphocytes (Fig. 4). Interestingly**,** patients who participated in the resistance exercise protocol exhibited a significantly reduced expression of both CD73 (*p* = 0.0001) and CD39 (*p* = 0.0001) on lymphocytes.

## Discussion

This study observed increased activity of NTPDases, ecto-5′-nucleotidases, and ADA in platelets and decreases in extracellular ATP levels, revealing a purinergic signaling imbalance in CKD. After resistance exercise, the hydrolysis of both ATP and AMP decreased and extracellular ATP levels were restored. Flow cytometry revealed that CKD patients present high CD39 and CD73 expression, and resistance exercise reduced the expression of both CD39 and CD73 enzymes in lymphocytes of these patients. Importantly, over 90% of our CKD patients presented hypertension, and the resistance exercise protocol significantly reduced their systolic blood pressure, suggesting an anti-hypertensive effect. Collectively, our findings support that resistance physical exercise performed for 12 weeks mitigates purinergic imbalance and presents a cardioprotective role in patients with CKD.

Cardiovascular disease is the leading cause of premature death in individuals with CKD, and this elevated risk goes beyond traditional factors like hypertension, diabetes, and dyslipidemia [27]. Non-traditional factors specific to CKD—particularly chronic inflammation, oxidative stress, and the accumulation of uremic toxins [29, 30]–—may contribute significantly by impairing vascular and cardiac function. Additionally, CKD-related mineral and bone disorder, anemia, and altered lipid metabolism further promote vascular calcification and left ventricular hypertrophy [31, 32]. These findings highlight the multifactorial nature of cardiovascular risk in CKD. In general, physical activity provides protective effects in CKD beyond managing traditional cardiovascular risk factors. Regular exercise reduces systemic inflammation, improves vascular function, and may enhance the clearance of uremic toxins and oxidative stress [29, 33]. Beneficial effects make exercise a valuable complementary strategy to mitigate both traditional and non-traditional cardiovascular risks in CKD patients.

IGF-1 is an anabolic hormone which is an important mediator of growth hormone and signaling pathways [34]. The main functions of IGF-1 include the regulation of cellular metabolism, proliferation, growth, and apoptosis, as well as anabolic actions in adults [35]. IGF-1 binds to its receptor and recruits the signalization of phosphoinositide 3-kinase (PI3K), producing the phosphorylation of Akt. The PI3K/Akt pathway plays a critical role in muscle fiber hypertrophy [36]. In summary, IGF/Akt controls two protein synthetic pathways via mTOR and glycogen synthase kinase-3β (GSK3β) pathway [37]. Studies involving healthy controls have demonstrated higher IGF-1 levels compared to those observed in our CKD patients [38–42]. Resistance exercise stimulated a slight but significant increase in IGF-1 in CKD patients. However, after exercise, values remained lower than those in healthy controls. In general, reduced IGF-1 levels in CKD may be associated with the degree of sarcopenia often present in these patients. Similarly to our findings, a meta-analysis highlighted that IGF-1 was increased after resistance exercise specifically in patients beyond 40 years [37].

Myostatin is made of skeletal myofibers and acts by limiting muscle growth [43]. Regarding myostatin activity, this molecule exhibits all the characteristics of a chalone, which is a term used to describe molecules that function as tissue-specific growth inhibitors [43]. Sarcopenia, commonly referred to as “muscle loss,” is a prevalent condition among patients with CKD. It reflects a pathophysiological state of CKD, characterized by an increased catabolic protein environment and reduced muscle mass synthesis [14]. Here, patients performed a 12-week (3 times a week) resistance exercise. In general, the World Health Organization determined minimum recommendations (i.e., > 150 min/week of moderate-intensity physical activity or 75 min/week of vigorous-intensity physical activity or a combination of both) [44]. Regarding myostatin levels, we observed that healthy controls exhibited slightly higher levels of this marker compared to our CKD patients [45–48]. Furthermore, exercise significantly reduced myostatin levels in the CKD group. Importantly, this study has limitations. Since IGF-1 and myostatin were not measured in our healthy controls, the values obtained from CKD patients before and after resistance exercise were compared with data from literature, and the conclusion is difficult. Thus, more studies are necessary to understand metabolic changes in CKD. Importantly, recent studies suggest that purinergic signaling may modulate muscle physiology by influencing the expression of growth factors such as IGF-1 and myostatin [49, 50]. For instance, extracellular ATP and its metabolites, through P2 and P1 receptor activation, may regulate muscle cell proliferation, increasing intracellular Ca^2+^ levels, thereby stimulating muscle hypertrophy [50].

Physical exercise is considered a nonpharmacological therapy to counteract hypertension [51]. The beneficial effects of resistance exercise for 6 months on reducing blood pressure were demonstrated in hypertensive women [14]. In addition, a 12-week aerobic exercise program reduced 24-h and daytime ambulatory blood pressure as well as office blood pressure in patients with resistant hypertension [52]. In general, both resistance exercise and aerobic exercise training are effective to reduce blood pressure [12, 51]. Similarly to previous data, our findings highlighted that resistance exercise for 12 weeks reduces systolic blood pressure in patients with CKD. It is well known that physical exercise activates adaptive mechanisms by improving endothelial function, increasing nitric oxide (NO) production, stimulating pro-angiogenic pathways, and insulin sensitivity [53]. Previously, 12 weeks of resistance exercise reduced glucose levels in patients with CKD, confirming its benefits [23]. Furthermore, physical activity modulates the system-renin-angiotensin-aldosterone (SRAA) by reducing aldosterone and angiotensin II activity [53]. In addition, sympathetic nervous system activity (norepinephrine) was reduced after physical exercise [53]. Reducing blood pressure is especially important because around 92% of our CKD patients present systemic arterial hypertension. In this way, we suggest that physical exercise has a pleiotropic role and might regulate blood pressure, presenting protective cardiovascular responses in individuals with CKD.

Purinergic molecules (ATP, ADP, AMP, ADA, ectonucleotidases) and receptors (P2Y and P2X) are ubiquitously present in different parts of the body and play an important role in diseases mediating immune responses [5]. Here, patients with CKD show an increased activity of NTPDase/ATP, NTPDase/ADP, ecto-5′-nucleotidase/AMP, and ADA when compared to healthy controls. Interestingly, 12 weeks of resistance training decrease NTPDase/ATP activities in platelets. Resistance exercise (12 weeks) performed by patients with CKD results in the decrease of proinflammatory cytokines (IL-4 and IL-6) [15]. In general, while acute physical exercise may trigger tissue damage and inflammatory responses, chronic physical exercise exerts anti-inflammatory and anti-platelet effects [54]. Moreover, NTPDase plays an important role in regulating endothelial homeostasis and can coordinate platelet aggregation [55, 56]. Thereby, our data is indicative that exercise mitigates the alterations in purinergic signaling associated with CKD.

Increased hydrolysis of extracellular ATP by NTPDases, particularly CD39, may contribute to the reduced extracellular ATP levels observed in CKD patients. Our data are in accordance with previous studies which showed that CD39 and CD73 activity and overexpression result in decreased extracellular ATP levels [57, 58]. Interestingly, resistance exercise restored extracellular ATP levels to values similar to those of the control group, suggesting a modulatory effect of exercise. Purinergic signaling is known to contribute to renal homeostasis, including the regulation of water and electrolyte transport across all nephron segments [41]. However, this system is also implicated in kidney diseases, including nephritis, polycystic kidney disease, diabetes, hypertension, and nephrotoxic injury [41]. The combination of reduced extracellular ATP levels and increased activity of NTPDase/CD39 and ecto-5′-nucleotidase observed here may reflect a purinergic imbalance associated with CKD.

**Fig. 5:**
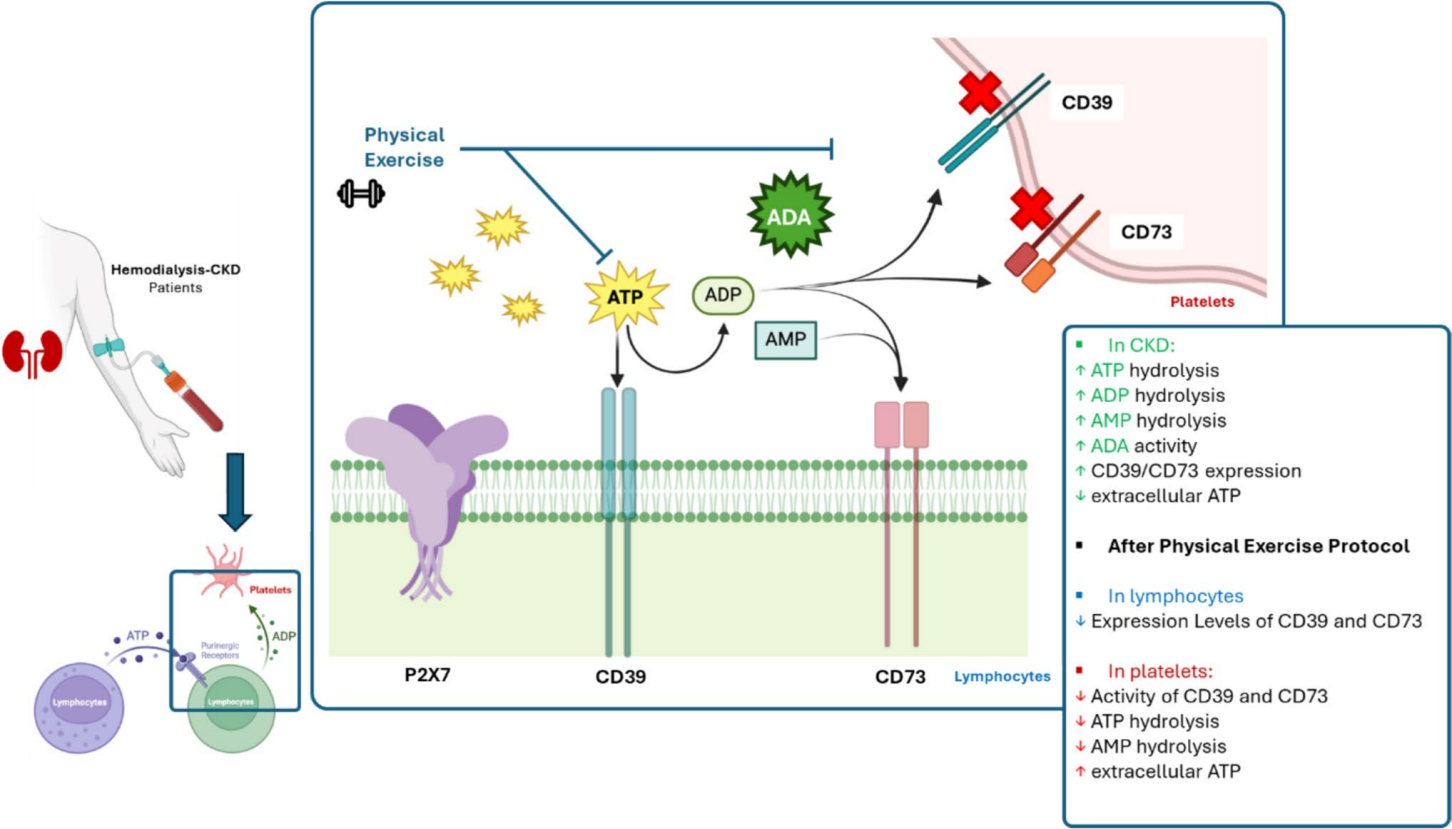
Patients with CKD exhibit increased ectonucleotidases’ activity and reduced extracellular ATP levels. A 12-week resistance training program modulates the activity and expression of the ectonucleotidases CD39 and CD73, as well as extracellular ATP levels in these patients

In general, CD39 expression is altered in numerous pathologic conditions, including autoimmune diseases, cancer, and atherosclerosis [59–61]. CD39 expression may be regulated by inflammatory cytokines, such as IL-6, leading to elevated CD39 levels [62]. In this study, both CD39 and CD73 expression were increased in patients with CKD, reflecting enhanced activity of the ectonucleotidases in platelets. Importantly, high NTPDase mRNA expression is associated with increased nucleotide hydrolysis [63]. Interestingly, resistance exercise reduced NTPDase/CD39 and ecto-5′-nucleotidase/CD73 density in lymphocytes. It is well known that both CD39 and CD73 are expressed in CD4^+^ and CD8^+^ T cells and help orchestrate immune responses [64]. The absence of a control group of healthy exercisers is a limitation and would have been valuable for a better understanding of the effects of exercise in both health and disease; thus, more studies are needed to clarify this point. However, our results support the idea that resistance exercise modulates purinergic signaling, specifically targeting CD39 and CD73 activities. Our findings are summarized in Fig. 5. In addition to purinergic endpoints, physical activity results in reduced systolic blood pressure. Together, these findings suggest that resistance exercise has a cardioprotective role and should be encouraged, particularly in patients with CKD.

## Conclusions

In conclusion, our findings provide important insights into the alterations in purinergic signaling associated with CKD. We demonstrated that both CD39 and CD73 activity and expression were elevated in CKD patients compared to healthy controls, while extracellular ATP was decreased, suggesting a compensatory effect. Our findings suggest that exercise modulates the activity and expression of CD39 and CD73 enzymes and the levels of extracellular ATP, thereby mitigating the alterations in purinergic signaling associated with CKD. Additionally, resistance exercise performed during hemodialysis sessions led to a reduction in blood pressure, likely mediated by adaptive physiological mechanisms. Overall, resistance exercise appears to have anti-hypertensive effects and should be encouraged in patients with CKD undergoing hemodialysis.

## Supplementary Information

Below is the link to the electronic supplementary material.

Supplementary file 1(PNG 432 KB)

High Resolution Image (TIF 869 KB)

Supplementary file 2(PNG 116 KB)

High Resolution Image (TIF 133 KB)

Supplementary file 3(PNG 191 KB)

High Resolution Image (TIF 251 KB)

Supplementary file 4(PNG 393 KB)

High Resolution Image (TIF 540 KB)

Supplementary file 5 (DOCX 13.4 KB)

Supplementary file 6 (DOCX 24.9 KB)

Supplementary file 7 (DOCX 25.2 KB)

## Data Availability

No datasets were generated or analysed during the current study.
