# Supplementary figures and images for: Resistance exercise exerts anti-hypertensive effects and downregulates NTPDase/CD39 and ecto-5′-nucleotidase/CD73 expression in patients with chronic kidney disease undergoing hemodialysis
Source: Purinergic Signal. 2026 Jan 21;22(1):11. doi: 10.1007/s11302-025-10121-7 (PMC12824041; doi:10.1007/s11302-025-10121-7)

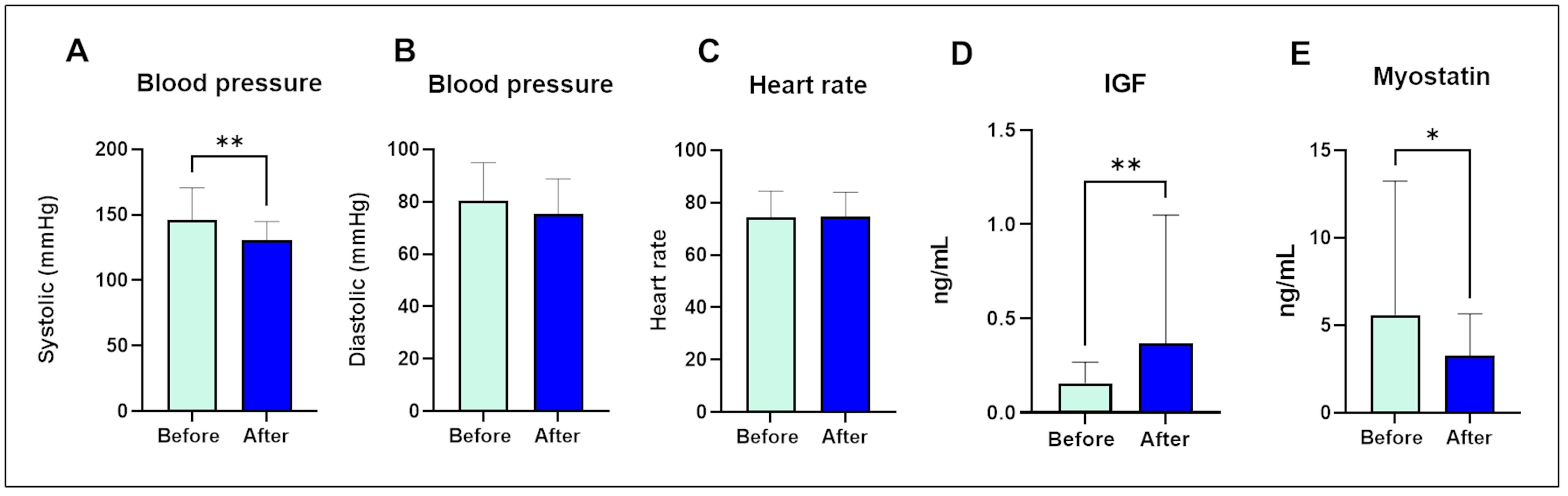

Supplement: Supplementary file 1 — (PNG 432 KB) [file 11302_2025_10121_Fig6_ESM.png]

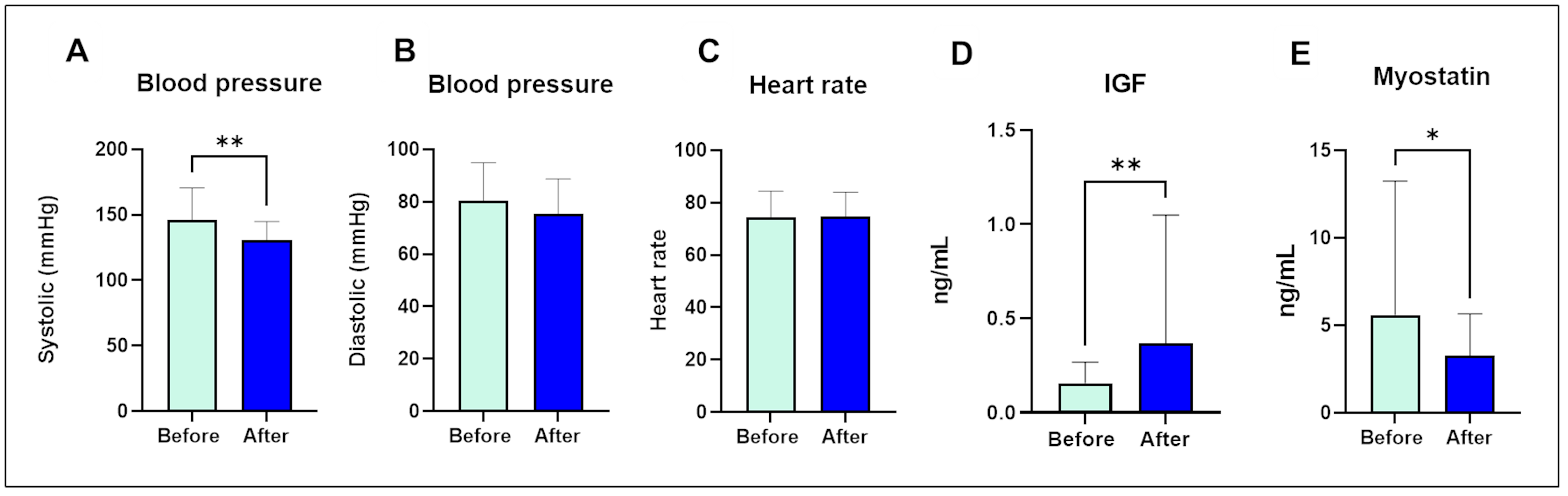

Supplement: Supplementary file 2 — High Resolution Image (TIF 869 KB) [file 11302_2025_10121_MOESM1_ESM.tif]

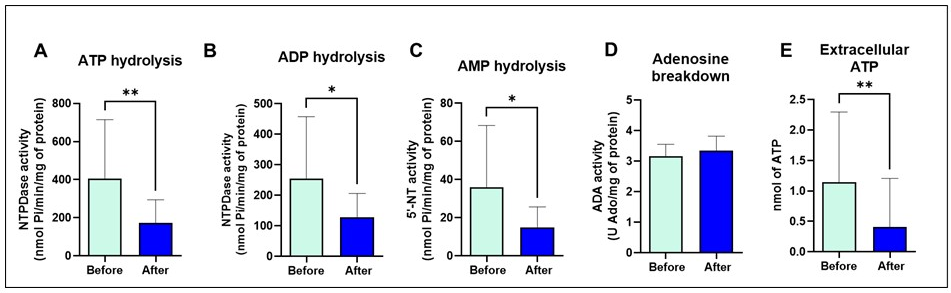

Supplement: Supplementary file 3 — Supplementary file 2(PNG 116 KB) [file 11302_2025_10121_Fig7_ESM.png]

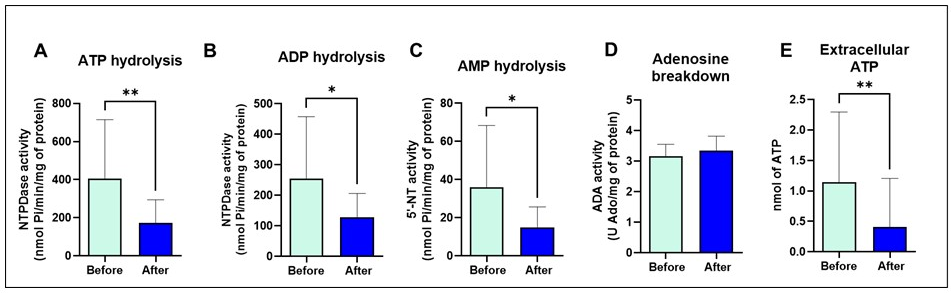

Supplement: Supplementary file 4 — High Resolution Image (TIF 133 KB) [file 11302_2025_10121_MOESM2_ESM.tif]

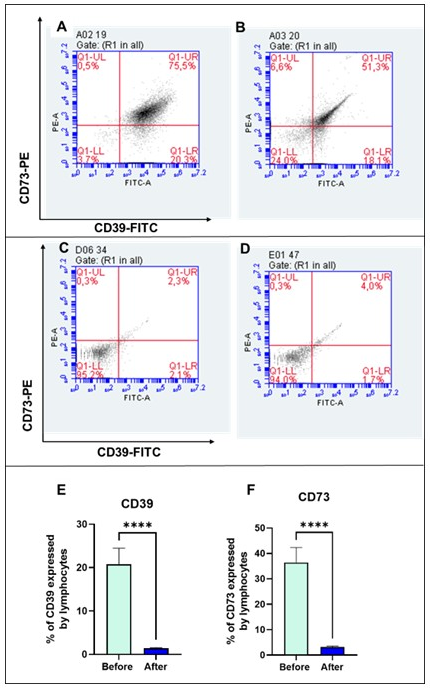

Supplement: Supplementary file 5 — Supplementary file 3(PNG 191 KB) [file 11302_2025_10121_Fig8_ESM.png]

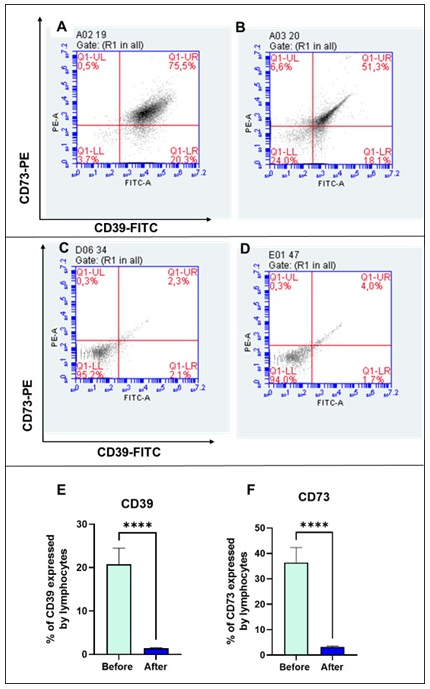

Supplement: Supplementary file 6 — High Resolution Image (TIF 251 KB) [file 11302_2025_10121_MOESM3_ESM.tif]

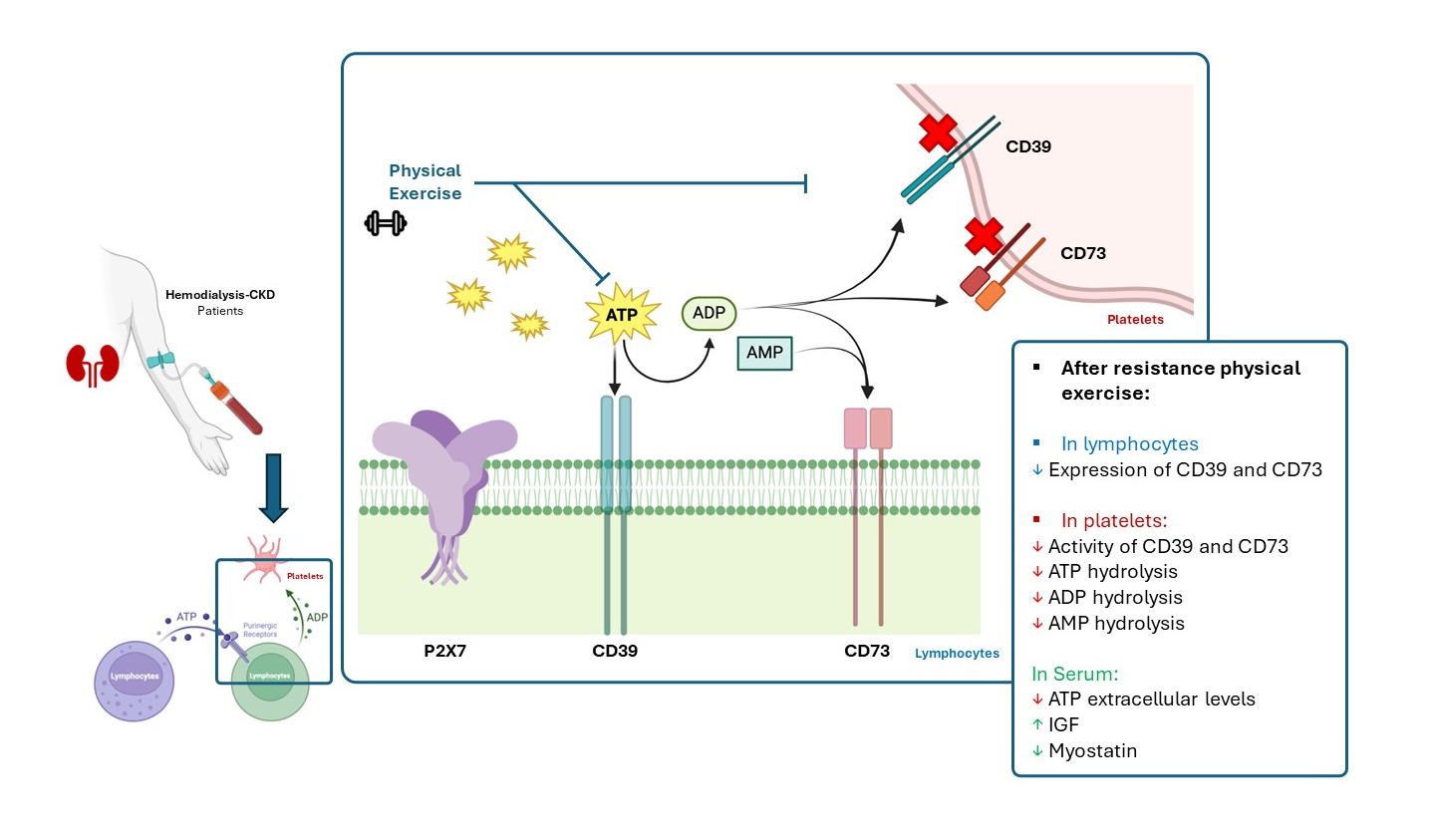

Supplement: Supplementary file 7 — Supplementary file 4(PNG 393 KB) [file 11302_2025_10121_Fig9_ESM.png]

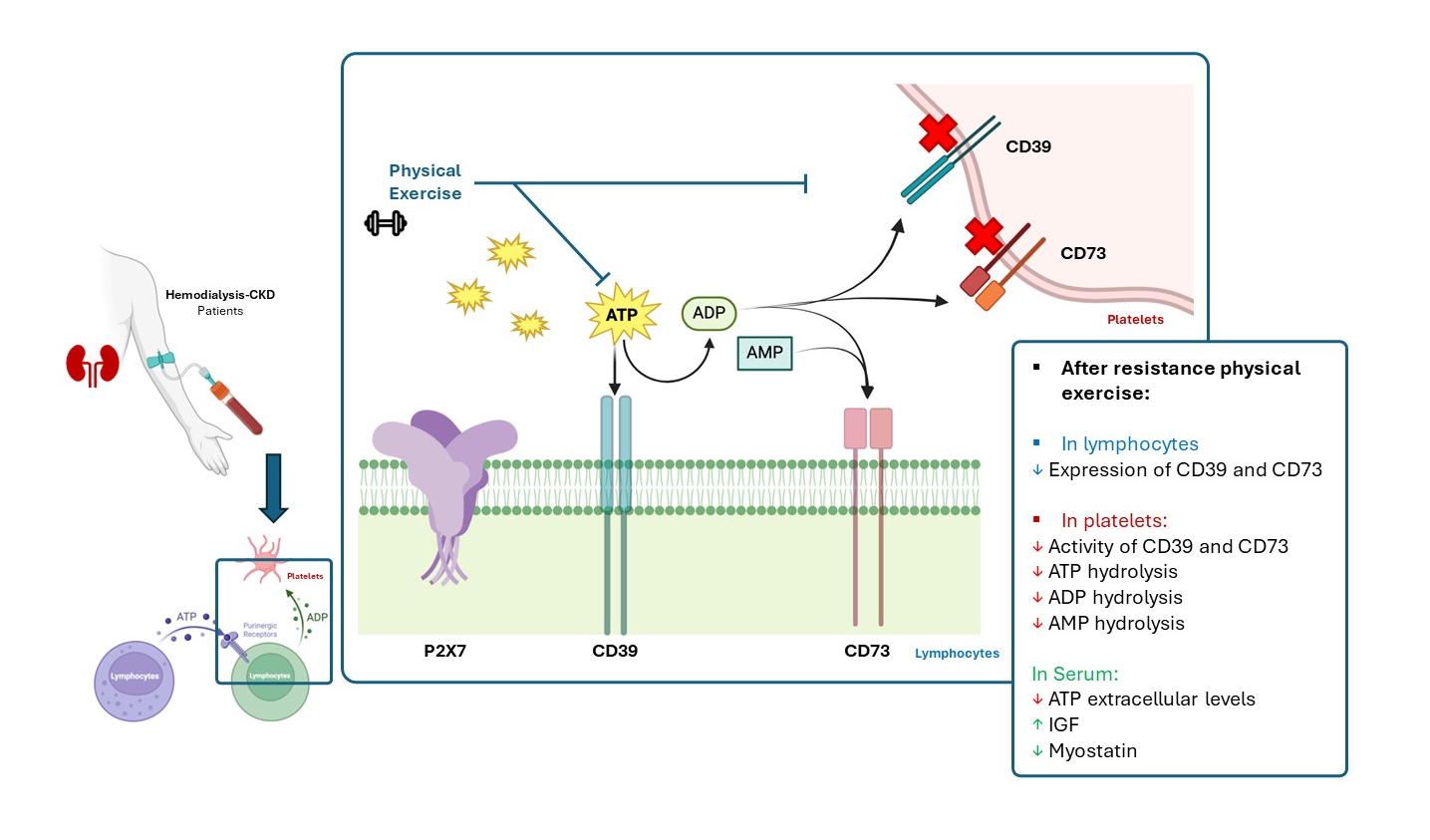

Supplement: Supplementary file 8 — High Resolution Image (TIF 540 KB) [file 11302_2025_10121_MOESM4_ESM.tif]
