# Supplementary material for: Resistance exercise exerts anti-hypertensive effects and downregulates NTPDase/CD39 and ecto-5′-nucleotidase/CD73 expression in patients with chronic kidney disease undergoing hemodialysis
Source: Purinergic Signal. 2026 Jan 21;22(1):11. doi: 10.1007/s11302-025-10121-7 (PMC12824041; doi:10.1007/s11302-025-10121-7)
Supplement: Supplementary file 9 — Supplementary file 5 (DOCX 13.4 KB) [file 11302_2025_10121_MOESM5_ESM.docx]

**Figure captions**

**Figure 1.** Blood pressure and metabolic endpoints. (**A**) Systolic blood pressure. (**B**) Diastolic blood pressure. (**C**) Heart rate. (**D**) Insulin like-growth-factor (IGF-1). (**E**) Myostatin. Data were expressed as median and interquartile range. (*) Indicates a significant difference *p≤0.05, **p≤0.01. (n = 19 - 27).

**Figure 2.** Purinergic enzyme activities analyzed in platelets of patients with CKD submitted to the physical exercise protocol. In platelets, NTPdase/CD39 activity in relation to ATP hydrolysis (**A**), NTPdase/CD39 activity in relation to ADP hydrolysis (**B**), ecto-5'-nucleotidase/CD73 activity in relation to AMP hydrolysis (**C**), ADA activity (**D**). Serum analyzes includes extracellular ATP (**E**). Data were expressed as median and interquartile range. The results were analyzed by the Mann-Whitney test. (*) Indicates a statistically significant differences *p≤0.05, **p≤0.01 and ***p≤0.001, ****p≤0.0001 (n= 19 - 28).

**Figure 3.** Representative flow cytometry dot-plots of patients with CKD submitted to resistance physical exercise. CD39 and CD73 expression in lymphocytes of patients before physical exercise (**A)** and (**B**). CD39 and CD73 expression after resistance physical exercise protocol (**C**) and (**D**). CD39 expression (**E**). CD73 expression (**F**). Dara were expressed as median and interquartile range. (*) Indicates a significant difference ****p≤0.0001. (n = 26 - 23).

**Figure 4.** Cardioprotective effects of resistance physical training in patients with CKD on hemodialysis. Resistance physical exercise for 12 weeks modulates purinergic enzyme activity and expression presents metabolic responses in patients with CKD.
