# Supplementary material for: Resistance exercise exerts anti-hypertensive effects and downregulates NTPDase/CD39 and ecto-5′-nucleotidase/CD73 expression in patients with chronic kidney disease undergoing hemodialysis
Source: Purinergic Signal. 2026 Jan 21;22(1):11. doi: 10.1007/s11302-025-10121-7 (PMC12824041; doi:10.1007/s11302-025-10121-7)
Supplement: Supplementary file 10 — Supplementary file 6 (DOCX 24.9 KB) [file 11302_2025_10121_MOESM6_ESM.docx]

| **Sample characterization** | | | | | | | | | | |  |
| --- | --- | --- | --- | --- | --- | --- | --- | --- | --- | --- | --- |
|  | | | | | | | | | | |  |
| **Control Patients** |  | | |  |  |  | |  | | |  |
| Total of sample | Group = 17 | | |  |  |  | |  | | |  |
|  |  | | |  |  |  | |  | | |  |
| ***Sex*** | ***Nº*** | ***%*** | | ***BMI*** | ***< 18.4*** | ***18.5 – 24.9*** | ***25 – 29.9*** | | ***>30*** | |  |
|  |  | |  |  |  |  | |  | | |  |
| *Male* | 7 | | 41% | 30.68 | 0 | 3 | | 2 2 | | |  |
|  |  | | |  |  |  | |  | | |  |
| *Female* | 10 | | 59% | 27.10 | 0 | 3 | | 5 2 | | |  |
|  |  | |  |  |  |  | |  | | |  |
|  |  | |  |  |  |  | |  | | |  |
|  |  | | |  |  |  | |  | | |  |
|  |  | | | ***Age*** | ***Smokers /*** | ***Alcoholics*** | |  | | |  |
|  |  | | |  | *No* | *Not informed* | |  | | |  |
| *% of Male* |  | | | 47.67 ± 7.28 | 7 (100 %) | - | |  | | |  |
| *% of Famale* |  | | |  |  | - | |  | | |  |
|  |  | | | 37.29 ± 10.37 | 10 (100 %) |  | |  | | |  |
|  |  | | | ***% of total*** | **17 (100 %)** | **-** | |  | | |  |
|  |  | | |  |  |  | |  | | |  |
|  |  | | |  |  |  | |  | | |  |
| ***Comorbidities*** |  |  | | ***Hypertension*** | ***DM II*** | ***Hypothyroidism*** | | ***Hyperthyroidism*** | | |  |
|  |  | | |  |  |  | |  | | |  |
|  |  |  | | 3 (17.64 %) | 1 (5.88 %) | - | | - | | |  |
| ***Medicine*** |  |  | | ***Anti-hypertensive*** | ***CNS***  ***medications*** | ***Hypoglycemic*** | |  | | |  |
|  | | | |  |  |  | |  | | |  |
| *% of Male* | | | | 1 (5.88 %) | - | 1 (5.88 %) | |  | | |  |
| *% of Female* | | | | 2 (11.76 %) | 3 (17.64 %) | - | |  | | |  |
|  | | | |  |  |  | |  | | |  |
| ***% of total*** | | | | **3 (17.64 %)** | **3 (17.64 %)** | **1 (5,88 %)** | |  | | |  |
|  |  | | |  |  |  | |  | | |  |
| **CKD Patients** |  | | |  |  |  | |  | | |  |
| Total of sample | Group = 28 | | |  |  |  | |  | | |  |
|  |  | | |  |  |  | |  | | |  |
| ***Sex*** | ***Nº*** | ***%*** | | ***BMI*** | ***< 18.4*** | ***18.5 – 24.9*** | ***25 – 29.9*** | | ***>30*** | |  |
| *Male* | 14 | 50% | | 27.5 | 0 | 6 | | 2 | | 6 | |
| *Female* | 14 | 50% | | 23.9 | 1 | 5 | | 8 | 0 | |  |
|  |  |  | |  |  |  | |  |  | |  |
|  |  |  | | ***Age*** | ***Smokers /*** | ***Alcoholics*** | |  |  | |  |
|  |  |  | |  | *No* | *Not informed* | |  |  | |  |
|  |  |  | |  |  |  | |  |  | |  |
| *% of Male* |  |  | | 49.15 ± 15.42 | 8 (54.14 %) | 6 (42.85 %) | |  |  | |  |
| *% of Female* |  |  | | 50.21 ± 18.50 | 9 (64.28 %) | 5 (35.71 %) | |  |  | |  |
|  |  |  | |  |  |  | |  |  | |  |
| ***% of total*** |  |  | |  | **17 (60.71 %)** | **11 (39.28 %)** | |  |  | |  |
|  |  |  | |  |  |  | |  |  | |  |
| ***Comorbidities*** |  |  | | ***Hypertension*** | ***DM II*** | ***Hypothyroidism*** | | ***Hyperthyroidism*** | | |  |
|  |  |  | |  |  |  | |  | | |  |
|  |  |  | | 26 (92.85 %) | 7 (25 %) | 1 (3.57 %) | | 1 (3.57 %) | | |  |
|  |  |  | |  |  |  | |  | | |  |
| ***Medicine*** |  |  | | ***Anti-hypertensive*** | ***Insulin*** | ***CNS medications*** | | ***Analgesic*** | | |  |
|  | | | |  |  |  | |  | | |  |
| *% of Male* | | | | 14 (100 %) | 4 (28.57 %) | 10 (71.42 %) | | 7 (50.00 %) | | |  |
| *% of Female* | | | | 12 (85.71 %) | 3 (21.42 %) | 10 (71.42 %) | | 7 (50.00 %) | | |  |
|  | | | |  |  |  | |  | | |  |
| ***% of total*** | | | | **26 (92.85 %)** | **7 (25 %)** | **20 (71.42 %)** | | **14 (50.00 %)** | | |  |
|  | | | |  |  |  | |  | | |  |
|  | | | | ***Levothyroxine*** | ***Iron-hydroxide*** | ***Erythropoietin*** | | ***Gastrointestinal medications*** | | |  |
|  | | | |  |  |  | |  | | |  |
| *% of Male* | | | | 0 (0 %) | 14 (100%) | 14 (100%) | | 13 (92.86%) | | |  |
| *% of Female* | | | | 4 (28.57 %) | 14 (100 %) | 14 (100%) | | 13 (92.86%) | | |  |
|  | | | |  |  |  | |  | | |  |
| ***% of total*** | | | | **4 (14.3 %)** | **28 (100 %)** | **28 (100 %)** | | **26 (92.86 %)** | | |  |
|  | | | |  |  |  | |  | | |  |
|  | | | | ***Statins*** |  |  | |  | | |  |
|  | | | |  |  |  | |  | | |  |
| *% of Male* | | | | 1 (7.14 %) |  |  | |  | | |  |
| *% of Female* | | | | 1 (7.14 %) |  |  | |  | | |  |
|  | | | |  |  |  | |  | | |  |
| ***% of total*** | | | | **2 (7.14 %)** |  |  | |  | | |  |
|  | | | |  |  |  | |  | | |  |
| ***Creatinine mg/dL*** | | | |  |  |  | | | | |  |
|  | | | | **Before** | **After** |  | |  | | |  |
| *Male* | | | | 11.23 ± 2.67 | 8.99 ± 2.61 * | | |  | | |  |
| *Female* | | | | 8.881 ± 2.742 | 8.053 ± 3.071 | | |  | | |  |
| *Total* | | | | 10.19 ± 2.85 | 8.411± 2.72 * |  | |  | | |  |
