# Supplementary material for: Resistance exercise exerts anti-hypertensive effects and downregulates NTPDase/CD39 and ecto-5′-nucleotidase/CD73 expression in patients with chronic kidney disease undergoing hemodialysis
Source: Purinergic Signal. 2026 Jan 21;22(1):11. doi: 10.1007/s11302-025-10121-7 (PMC12824041; doi:10.1007/s11302-025-10121-7)
Supplement: Supplementary file 11 — Supplementary file 7 (DOCX 25.2 KB) [file 11302_2025_10121_MOESM7_ESM.docx]

**Table 1.** Serum IGF-1 and myostatin levels of patients with CKD and healthy controls.

| **Group** | **IGF-1 (ng/mL)** | **Number of individuals** | **Reference** |
| --- | --- | --- | --- |
| Ctrl | 136.4 ± 47.4 | 62 | [1] |
| Ctrl | 147.08 ± 4.04 | 13 | [2] |
| Ctrl | 151.77±42.92 | 46 | [3] |
| Ctrl | 128.23 ± 25.83 | 60 | [4] |
| CKD (before) | 0.12 ± (0.08 – 0.18) | 28 | **-** |
| CKD (after) | 0.17 ± (0.14 – 0.32) ** | 28 | - |
| **Group** | **Myostatin (ng/mL)** | **Number of individuals** | **Reference** |
| Ctrl | 10.97 ± 6.77 | 52 | [5] |
| Ctrl | 7.46 ± 2.21 | 60 | [6] |
| Ctrl | 4.74 ± 1.25 | 142 | [7] |
| Ctrl | 3.9 ± (2 – 7) | 249 | [8] |
| CKD (before) | 3.70 ± (2.8 – 4.2) | 28 | - |
| CKD (after) | 2.72 ± (1.75 – 3.8) * | 28 | - |

Data were analyzed by Mann-Whitney test and results are expressed as median and interquartile range. *Indicates significative differences when compared patients with CKD before exercise. * p < 0.05; ** p <0.001.

**References**

1. Gironi M, Solaro C, Meazza C, et al (2013) Growth Hormone and Disease Severity in Early Stage of Multiple Sclerosis. Mult Scler Int 2013:836486. https://doi.org/10.1155/2013/836486

2. Torres-Aleman I, Barrios V, Berciano J (1998) The peripheral insulin-like growth factor system in amyotrophic lateral sclerosis and in multiple sclerosis. Neurology 50:772–776. https://doi.org/10.1212/WNL.50.3.772

3. Nageeb RS, Hashim NA, Fawzy A (2018) Serum insulin-like growth factor 1 (IGF-1) in multiple sclerosis: relation to cognitive impairment and fatigue. The Egyptian Journal of Neurology, Psychiatry and Neurosurgery 54:25. https://doi.org/10.1186/s41983-018-0026-y

4. Qiao X, Yan J, Zang Z, et al (2024) Association between IGF-1 levels and MDD: a case-control and meta-analysis. Front Psychiatry 15:. https://doi.org/10.3389/fpsyt.2024.1396938

5. de Sordi CM, dos Reis-Neto ET, Keppeke GD, et al (2022) Serum Myostatin and Follistatin Levels in Patients With Dermatomyositis and Polymyositis. JCR: Journal of Clinical Rheumatology 28:33. https://doi.org/10.1097/RHU.0000000000001806

6. Ju C-R, Chen R-C (2012) Serum myostatin levels and skeletal muscle wasting in chronic obstructive pulmonary disease. Respiratory Medicine 106:102–108. https://doi.org/10.1016/j.rmed.2011.07.016

7. Li W, Yin R, Xia X, et al (2025) Correlations of serum myostatin and irisin with sarcopenia and osteoporosis in rheumatoid arthritis patients: a cross-sectional study. Sci Rep 15:23068. https://doi.org/10.1038/s41598-025-07378-8

8. Breitbart A, Scharf GM, Duncker D, et al (2013) Highly specific detection of myostatin prodomain by an immunoradiometric sandwich assay in serum of healthy individuals and patients. PLoS One 8:e80454. https://doi.org/10.1371/journal.pone.0080454
